# Breast cancer on social media: a quali-quantitative study on the credibility and content type of the most shared news stories

**DOI:** 10.1186/s12905-021-01352-y

**Published:** 2021-05-15

**Authors:** Priscila Biancovilli, Lilla Makszin, Alexandra Csongor

**Affiliations:** 1 Doctoral School of Health Sciences, University of Pécs, Pécs, Hungary; 2 Institute of Bioanalysis, Medical School, University of Pécs, Pécs, Hungary; 3 Szentágothai Research Center, University of Pécs, Pécs, Hungary; 4 Department of Languages for Biomedical Purposes and Communication, University of Pécs, Medical School, Pécs, Hungary

**Keywords:** Breast cancer, Social media, Social networking sites, Content analysis, Credibility, Misinformation, Prevention, Early detection

## Abstract

**Background:**

Female breast cancer was the most diagnosed cancer in 2020, with more than two million new cases worldwide. Access to scientifically correct information can assist patients in early detection or prevention of the disease. However, misinformation on social networking sites (SNSs) about breast cancer can be propagated rapidly, posing a threat to health communication efforts. The aim of this study is to analyse the characteristics of the most shared news stories referencing the disease that circulated on SNSs, including the credibility of this content.

**Methods:**

This is an exploratory quali-quantitative study. Data collection was conducted between June 2019 and June 2020. We performed statistical and content analysis of the stories that had at least 1,000 total shares. Each story was classified in accordance to the following aspects: credibility; type of rumour; source; content type; mentions prevention or early detection/screening exams.

**Results:**

The abundance of news stories in our sample (n = 1,594) were not classified according to their credibility, as they do not address science, risk factors, prevention, treatment, or other aspects which can be assessed for scientific accuracy. However, content classified as “rumours” are 3.29 times more shared than those considered scientifically correct. Regarding content type, most stories are classified as ‘real-life story’ or ‘solidarity’ (67.69%). In our sample, 5.08% of the total comment on prevention and 19.7% reference early detection.

**Conclusion:**

We consider it can be a good strategy, in SNSs, to combine content of greater popularity, such as real-life stories, with subjects that can make a difference in a patient’s life, such as early detection, breast cancer symptoms and disease prevention strategies. Doctors, scientists and health journalists can expand the dialogue with the lay public regarding breast cancer, helping to counteract online misinformation.

## Background

Breast cancer is one of the most frequently occurring types of cancer in the world; female breast cancer has surpassed lung cancer as the most commonly diagnosed cancer in 2020, with an estimated 2.3 million new cases worldwide [1]. Early detection is extremely important towards improving the survival rate of patients. Additionally, nearly 23% of breast cancer cases are preventable [2]. More than 90% of women diagnosed with breast cancer at the earliest stages (stages 0 and I) survive their disease for at least five years compared to around 15% for women diagnosed with the most advanced, metastatic stage of disease (stage IV) [3]. It is of fundamental importance patients and family caregivers understand the role of chemotherapy, radiotherapy and other conventional treatments for curing the disease [4]. For this reason, it is essential to develop efficient health communication strategies aimed at the lay public. October is Breast Cancer Awareness Month, an international health campaign which aims to increase awareness of the disease, including the importance of prevention, self-examination, screening and to raise funds for ongoing research [5].

In recent times, social networking sites (SNSs), which are web-based services that allow users to create a profile and connect with other individuals within the system, have emerged as powerful health communication platforms [6]. An increasing number of individuals rely on social networking as a source of information. In a worldwide survey conducted in 2020, more than 65% of respondents from populous countries such as Mexico, Argentina, Kenya, South Africa, Philippines and Brazil, declared they rely on social media as a source of news [7]. Moreover, 53% of EU citizens aged 16–74 reported they sought online health information related to injury, disease, nutrition, improving health or similar; in which the highest shares were recorded in Finland (76%) and in the Netherlands (74%) [8].

Despite its immense power to reach a wide audience, SNSs present some drawbacks. Users can share content without any verification by editors, reviewers, or fact-checkers. Often, such content reaches a number of users similar or even greater than traditional media [9]. This overabundance of information, both accurate and inaccurate, makes it more difficult for the lay audience to filter and learn essential information regarding a given subject.

A widely discussed topic in studies related to communication in the SNSs is the “fake news” phenomenon [10]. The Harvard Kennedy School’s Shorenstein Center defines fake news as “misinformation that has the trappings of traditional news media, with the presumed associated editorial processes” [11]. In numerous studies, nevertheless, this term has been replaced by others, since it is considered inadequate to capture the complexity of the information disorder phenomenon [12–14]. Terminologies used extensively in literature include misinformation, which is, false, inaccurate or misleading (out of context) information, regardless of whether there is intent to mislead [12, 15]; and disinformation, which entails the distribution, assertion, or dissemination of false, mistaken, or misleading information in an intentional, deliberate, or purposeful effort to mislead, deceive, or confuse [13, 16]. For the purpose of this study, the term ‘misinformation’ is used as an umbrella expression encompassing all characteristics of information which lack scientific evidence, since we are not able to know, in most cases, if the author had the deliberate intention of spreading false, misleading or confuse information.

In relation to health, social networks serve as fertile ground for the proliferation of misinformation. One can use as an example, Facebook, the SNS with the largest number of active users as of January 2021 [17]. A study analysed the credibility of the ten most popular health news stories featured on this social network [18] and found only three articles achieved a high credibility rating, and four articles received a medium credibility rating (in this case, the information is not entirely false, however, it contains misleading data). In April 2019, an article entitled "Cancer industry not looking for a cure; they're too busy making money," had nearly three million engagements on Facebook until it was banned on this SNSs for using misleading and inaccurate information [19].

Since wrong and malicious information can be quickly propagated on SNSs, efforts must be redoubled to better communicate medical advances accurately with both the lay public and among patients to ensure genuine knowledge can be separated from false material [20]. In two studies [21, 22], researchers investigated the relationship between the use of complementary and alternative medicine, adherence to conventional treatment, and overall survival among patients with cancer. Together, the studies revealed that patients who use alternative or complementary medicine are more than twice as likely to die when compared with those treated using conventional methods. Moreover, those patients are more likely to refuse surgery. This can be a consequence of a decline in public trust in physicians, generated by the increase of health and cancer misinformation spread over the internet [20].

The aim of this study is to analyse news stories about breast cancer shared on social networks from varied perspectives. We seek to understand the characteristics regarding the narratives in our sample, including the credibility of the content with more public engagement. Health professionals and communicators need to know the attributes of breast cancer-related content currently circulating among SNSs, in order to disseminate relevant and accurate content in an appealing way, ultimately counteracting misinformation. To our knowledge, despite the large number of studies regarding online health-related and cancer misinformation, this is the first investigation dedicated exclusively towards effectively analysing breast cancer content across the most used SNSs worldwide. Our research questions are:

- **RQ1.** What is the credibility of the most shared content?
- **RQ2.** What are the characteristics of the breast cancer news stories on social networks that generates more engagement (in the form of total shares)?
- **RQ3.** Do these stories address prevention or early detection in breast cancer?
- **RQ4.** Are there any differences between the content shared in October (Breast Cancer Awareness Month) and other months of the year?

## Methods

### Study design and data collection

This is an exploratory quali-quantitative study, without prior hypotheses. We analysed news stories in the English language regarding breast cancer. Data collection was conducted between 17 June 2019 and 17 June 2020.

To collect our sample, we used an online tool called Buzzsumo [23], which monitors the web and social media feeds to show the most popular content in any niche. We searched for the keyword "breast cancer", in quotation marks, so that we only have results displaying this exact term, and not the words separately. The search was made within the "Web Content" tab, which finds and analyses the most engaging articles and blogs among the following social media sites: Facebook, Twitter, Pinterest and Reddit. These social networks are among the most popular in the world. In 2020, Facebook has more than 2.7 billion monthly active users [24]; Twitter has 321 million [25]; Pinterest has 442 million [26], and Reddit, 430 million [27].

Our search was limited to pages in English, with no country restrictions. We performed statistical and content analysis of the stories which had at least 1,000 total shares. The sample size was determined based on the following: first, since most shared news stories were exactly those which had greater visibility throughout the studied social networks, and therefore these stories are more relevant to our investigation; secondly, we need to establish a cut for this sample, which makes content analysis possible; lastly, we believe this cut is enough to have a comprehensive overview of the conversations and narratives regarding breast cancer in the chosen period.

The news stories filtered by Buzzsumo were exported to an Excel table containing the following information: total shares (sum of shares across all analysed social networks); total Facebook shares; Twitter shares; Pinterest shares; total Reddit engagements; and published date.

### Content analysis

The content analysis follows the methodology developed by Bardin [28] (with some adjustments), an inductive process comprising of the following steps:

1. **Pre-analysis:** the researchers collect the *corpus* to be examined and implement a wide and careful reading of all relevant material.
2. **Coding:** coding is the step in transforming raw data from *corpus*, making use of records to be grouped in the future [29]. In this step, we developed the coding schedule for this research, which is, the form onto which all the data relative to the news stories being coded will be entered (as found in Table 1).
3. **Categorization:** each news story was considered by us as a unit of our *corpus*. In this way, we used different dimensions to categorize each column of the coding schedule (Table 1). Two researchers dedicated themselves to the analysis of the material and its classification. The coding schedule and its dimensions were previously established by both. Subsequently, the analysis of a sample of one hundred news stories was carried out separately by each of the researchers. Percent agreement was used to calculate inter-rater reliability, and the result is 83%. After analysing this initial sample, one of the researchers completed the categorization of the entire corpus.
4. **Interpretation:** after categorizing the entire corpus, we evaluated the results and made inferences.

**Table 1:** Coding manual, comprising the coding schedule (the column headings indicate the dimensions to be coded) and its categories

Credibility	Type of rumour	Source of news stories	Content type	Mentions prevention?	Mentions early detection or screening exams?
Verified	Misleading	Traditional media	Real-life story	Yes	Yes
Rumour	False connection/context	Digital media	Risk factors	No	No
	Fabricated content		Treatment		
	Satire		New technology		
			Solidarity		
			Educational		
			Complaint		
			Opinion		

### Credibility analysis and types of rumours

We initiated our content analysis by classifying news stories according to its credibility. For this, we first separated them into the following categories: "Verified" (scientifically accurate) and "Rumours" (scientifically inaccurate or false) [10]. To determine accuracy, the researchers checked whether the content of the news stories could be found in peer-reviewed journals indexed in the main health-related databases, which include the following: PubMed, PsycINFO, Embase, Cochrane Library, CINAHL and Web of Science [30]. It is important to mention not all stories were classified in “credibility” dimension; only those that address some kind of scientific innovation, traditional treatment, alternative treatments or mention health tips for patients, as well as address prevention and early detection in breast cancer.

For the “types of rumours” column, we were inspired in the nomenclature developed by Wardle [14] on the different types of misinformation, with some adaptations. The following categories were established: **(1) Misleading content:** describes stories which are not entirely false yet lead the reader to misinterpret the data; **(2) False connection/context:** this encompasses Wardle’s categories of manipulated content, false connection, and false context. We classified a rumour in this category when the headline does not support the content of the news story, or when genuine images, videos, photos, and audios were used outside their original context, or were manipulated. **(3) Fabricated content:** News stories without any trace of genuine information (both in the textual and non-textual parts) were classified in this category.

### Source of news stories

We classified the origin of news stories into three categories: (1) Traditional media: also known as ‘legacy media’ [31] or ‘old media’ [32], refers to the types of media that existed before the popularization of the internet, even though they now have their digital versions. As an example, we can mention radio or television networks, newspaper publishers, book publishers and movie studios. (2) Digital media: news sources launched online and exclusively publish using this medium [10].

### Content type analysis

The categories identified by the researchers for "content type" are as follows: (1) “Real life story”: testimonials from individuals who have/had cancer, or family members of patients, or other life stories; (2) “Risk factors”: stories whose main focus is on some risk factor for breast cancer, such as smoking, unhealthy eating habits, physical inactivity, among others; (3) “Treatment”: stories focused on explaining or announcing some type of treatment for breast cancer, whether they are traditional or alternative methods; (4) “New technology”: stories that focus on explaining new technologies in the detection or prevention of breast cancer; (5) “Solidarity”: stories that focus on solidarity actions, such as donating money to help women perform diagnostic tests, or when individuals become involved in breast cancer awareness actions, for example. (6) “Educational”: news stories that teach what kind of food can help prevent cancer, or what are the symptoms of breast cancer; (7) “Complaint”: reports of problems that breast cancer patients experience, such as the lack of medication or problems with health insurance providers; (8) “Opinion”: the focus is on the authors' opinions regarding topics related to breast cancer, such as awareness campaigns or new treatments.

### Mentions prevention and early detection/screening

Finally, we have included in our content analysis some questions whose answer is a simple 'yes' or 'no' (Table 1). The purpose of such questions is to evaluate in greater depth the content of news stories, seeking to understand how they approach breast cancer: whether they mention prevention strategies, early detection and screening exams.

### Statistical analyses

Data were analysed using IBM SPSS Statistics (version 26.0) predictive analysis software, and Microsoft Office Excel (version 16). Depending on the sample size, Fisher's Exact Test or Chi-square test was used to determine the relationship between two categorical variables. A Kolmogorov–Smirnov normality test was used to determine whether sample data has been drawn from a normally distributed population.

## Results

Screening the media for breast cancer news stories published between June 2019 and June 2020 resulted in 9,811 hits. Of these, 1,594 news stories had at least 1,000 total shares.

### Source of news stories

Most of the stories in our sample were published in digital media (76.73%), whereas 23.27% originated from traditional media. However, the most shared news story (Table 2) was published in Fox News, a traditional media outlet. Among the twenty stories with the most shares, thirteen were published by traditional media outlets: four times by Fox News and once by nine other media entities, such as The Epoch Times, Metro, CNN and NBC News. The most shared story in a digital media is authored by the blog The Breast Cancer Site.

**Table 2:** Top 20 most popular news stories related to breast cancer (measured by total shares in social networking sites), its credibility and content type, between June 2019 and June 2020

Rank	News story title and webpage	Total shares	Credibility: Verified or rumour	Content type
1	Trial vaccine wipes out breast cancer in Florida patient (Fox News Orlando)	1,822,993	Rumour	Treatment
2	Loyal boyfriend who stuck with girlfriend during breast cancer proposes on her last day of chemo (The Epoch Times)	734,482	n/a	Real-life story
3	Breast Cancer Vaccine Has Eliminated Cancer in Its First Human Patient (The Breast Cancer Site)	729,185	Rumour	Treatment
4	Dad with breast cancer 'rejected from support groups because he's a man' (Metro)	640,106	n/a	Real-life story
5	Albuquerque Police Department paints a patrol car pink for Breast Cancer Awareness Month (CNN)	635,438	n/a	Real-life story
6	Shannen Doherty reveals breast cancer is back, now stage 4 (NBC News)	356,393	n/a	Real-life story
7	Scientists Successfully Turn Breast Cancer Cells Into Fat to Stop Them From Spreading (Science Alert)	352,749	Verified	Treatment
8	Mom of 6 who survived breast cancer dies from COVID-19 (WGXA)	279,729	n/a	Real-life story
9	Trial vaccine wipes out breast cancer in Florida patient (Fox News Phoenix)	267,336	Rumour	Treatment
10	New blood test can detect breast cancer 5 years before lumps appear (NY Post)	188,567	Rumour	Treatment
11	Mom of 6 who survived breast cancer dies from COVID-19 in Snohomish Co. (KOMO-TV)	167,651	n/a	Real-life story
12	Olivia Newton-John winning breast cancer battle as tumours shrunk thanks to marijuana (Express)	164,526	Rumour	Treatment
13	Mom of 6 who survived breast cancer dies from COVID-19 (Local 12)	161,878	n/a	Real-life story
14	Trial vaccine wipes out breast cancer in Florida patient (Fox News DC)	161,035	Rumour	Treatment
15	19-year-old Ghanaian creates system to predict and diagnose breast cancer (Ghana Web)	149,237	Rumour	New technology
16	Black women are over 6 times more likely to get breast cancer from hair dye and relaxers- New study finds (Pulse)	145,238	Rumour	Risk factors
17	Mayo Breast Cancer Vaccine Could Be Available In Less Than A Decade (Forbes)	141,629	Verified	Treatment
18	Shannen Doherty shares she has stage 4 breast cancer: 'I'd rather people hear it from me' (Good Morning America)	138,195	n/a	Real-life story
19	Sad News, Robin Roberts Painfully Reveals She Had Breast Cancer. (YouTube)	134,769	n/a	Real-life story
20	Mom of 6 who survived breast cancer dies from COVID-19 (Fox News Nashville)	130,886	n/a	Real-life story

### Credibility and type of rumours

Regarding **RQ1**, of all news stories selected for coding, 69.7% have not been classified according to credibility. This is due to the fact these news items do not address science, risk factors, prevention, treatment or other aspects, which can be assessed for scientific accuracy. Among the news classified according to credibility (n = 483), 17.25% are 'verified' and 13.05% are 'rumours'.

When we examine the most common types of rumours, we see ‘false connection/context’ represent 62.7% of the total, ‘misleading content’ are 34.9% of the total, and totally false content, that is, 'fabricated content' category, represents 2.4% of the total.

**Fig. 1:**
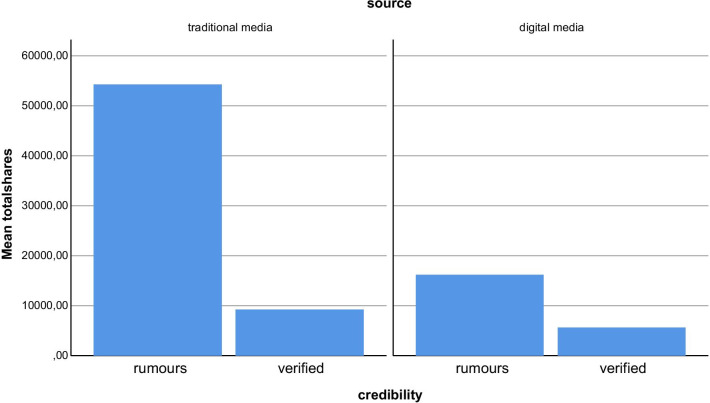
Mean of total shares in relation to the credibility of the content, separated by traditional media and digital media

In consideration of the number of shares in relation to the credibility of the content (Fig. 1), we see the content classified as "rumours" tends to be more shared than those scientifically correct, both in traditional and digital media. Although less frequent in our sample, “rumours” totalled 5,755,192 shares, whereas “verified” stories were tallied at 1,747,352 total shares (3.29 times less).

We can observe a very strong statistical connection (Cramer’s value = 0.313) between categories “credibility” and “content type”. News stories regarding “treatment” are 37.9% “rumours” and 62.1% (1.6 times more) “verified”. “Real life stories” are 58.3% (1.4 times more) “rumours” and 41.7% “verified”. About “risk factors”, 56.3% are “rumours” and 43.8% are “verified”; in “new technology”, 53.8% (nearly 1.5 times more) are “rumours” and 46.3% are “verified”. Stories classified as “educational” are 13.7% “rumours” and 86.3% (6.3 times more) “verified”. Finally, the dimensions “solidarity” and “complaint” are both 100% “verified” in our sample.

There is also a very strong association (Cramer’s value = 0.431) between categories “type of rumour” and “content type”. In the dimension “risk factors”, we observe that 79.5% of the rumours were classified as “false connection/context”, and 20.5% were deemed “misleading.” In the “treatment” dimension, 29.3% of the rumours are “misleading” and 70.7% (2.5 times more) have “false connection/context.”

### Content type

**Fig. 2:**
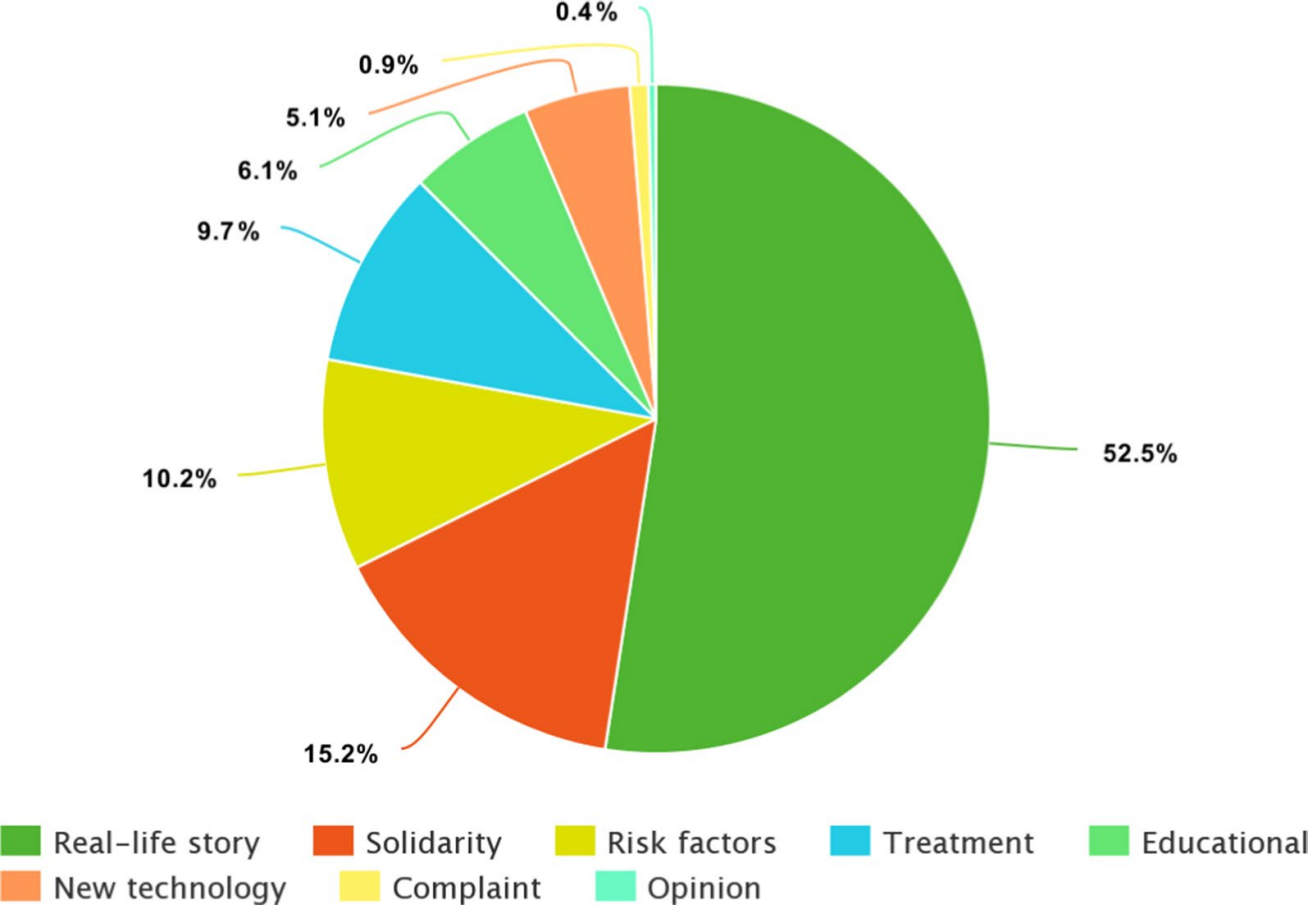
Percentage of news stories in each content type dimension

Regarding **RQ2**, when we examine the distribution of content type categories in our sample (Fig. 2), we see most stories are classified as ‘real-life story’ or ‘solidarity’ (67.69%). These stories have no scientific content, since they are focused on narrating the life of an individual or family members with cancer, publicizing actions to raise money for cancer hospitals or requesting donations of any kind for patients in need, to mention a few examples.

During the period studied, we also noticed a recurrence of the same news among the stories with the most total shares (Table 2). These stories multiply on different web pages, often with the same title and text, or few variations.

Among the most shared stories, we see how the trial vaccination against breast cancer of a patient in Florida was highlighted (the story was repeated four times in the Top Twenty). The death from COVID-19 of a mom of 6 who survived breast cancer was also noteworthy, being repeated four times.

We also noticed a highlight in news that addresses celebrities with breast cancer, including the North-American actress Shannen Doherty, the British-Australian singer Olivia Newton-John, and the North-American television broadcaster Robin Roberts.

### Mentions prevention and early detection/screening

To answer **RQ3**, most analysed news stories do not address ways of preventing or early detecting breast cancer (Table 3). In our sample, 5.08% of the stories comment on prevention and 19.7% mention early detection. There is an extraordinarily strong statistical connection (Cramer’s value = 0.435; Fisher’s exact test, *p* < 0.001) between content type and prevention; and between content type and early detection (Cramer’s value = 0.355; Chi-square test: *p* < 0.001).

**Table 3:** Percentage of news stories that address prevention and early detection, according to each content type dimension

	Prevention			Early detection		
	Yes	No	Total	Yes	No	Total
Treatment	0.6	99.4	100	17.5	82.5	100
Real-life story	0.7	99.3	100	16.6	83.4	100
Risk factors	25.3	74.7	100	5.6	94.4	100
New technology	1.2	98.8	100	73.2	26.8	100
Solidarity	1.2	98.8	100	15.2	84.8	100
Complaint	0	100	100	42.9	57.1	100
Educational	27.8	72.2	100	33	67	100
Opinion	33.3	66.7	100	66.7	33.3	100

Stories whose theme are "opinion", "educational" and "risk factors" have the highest proportion of references in prevention. In relation to early detection, we see this characteristic in stories regarding “new technology”, “opinion” and “complaint”. We did not observe statistical connection between prevention *versus* credibility and between early detection *versus* credibility.

### Breast cancer awareness month

Regarding **RQ4**, we compared the news published in October (known as the “Breast Cancer Awareness Month” or “Pink October” in several countries around the world [5]) with the other months. There are several variations in relation to the topics covered and in relation to the credibility of the most shared content (Chi-square test: *p* < 0.001; Cramer’s value = 0.300, extraordinarily strong connection).

There is a significant increase in news stories classified as "solidarity" in October (28.4% *versus* 9.3% in other months). On the other hand, there was a decrease in content that addresses “risk factors” (3.6% *versus* 13.1% in other months), “real-life stories” (47.9% *versus* 54.5% in other months) and “new technology” (1.0% *versus* 7.0% in other months). We can observe a slight increase in educational content (7.8% *versus* 5.3%).

When we compare the credibility of the news shared in October with the other months of the year, we see there is a statistically significant difference in the distribution of the types of rumours (Fisher’s exact test: *p* = 0.030; Cramer’s value = 0.172, strong connection). There is an increase in rumours classified as "false connection/context" (81.3% in October *versus* 59.3% in other months), whereas it is possible to note a decrease in “misleading content” (15.6% in October *versus* 38.4% in other months). There is no noteworthy difference in relation to "fabricated content" (3.1% in October *versus* 2.3% in other months).

We found a moderate connection (Chi-square test: *p* = 0.003; Cramer’s value = 0.138) between news stories’ credibility in October and in other months. Overall, in October there is an increase in news stories classified as “verified” (69.8% in October *versus* 53.3% in other months).

## Discussion

The aim of the present study was to analyse the most shared news stories regarding breast cancer on social networks, examining its content under different aspects. The majority (69.7%) of our sample could not be classified as to credibility, as they do not address topics related to science, technology and treatments. However, it is important to note that the news classified as "rumours" (13.05%) had a total number of shares 3.29 times greater than the "verified" ones (17.25%). This trend has previously been observed. In a study that evaluated the accuracy of the most popular articles on SNSs relating to genitourinary malignancies [33], there was a significantly higher average number of shares for inaccurate and misleading articles, compared to accurate ones. The same tendency was observed in a study dedicated to examine the spread of information related to Zika virus on the internet [10].

Most "rumours" in our sample did not display completely fabricated information, but instead presented “false connection/context” (62.7%) or “misleading content” (34.9%). Understanding this nuance regarding misinformation about breast cancer on social media is important. Valid information taken out of context can have even greater potential damage, as it may seem far more convincing to the lay reader—hence the higher number of total shares, which was also observed in other studies [34, 35].

To cite an example, we mention the most shared news story in our sample, entitled “Trial vaccine wipes out breast cancer in Florida patient” (1,822,993 total shares). It was classified as ‘false connection/context’ because the title implies the vaccine is a reality, since a patient has been cured of cancer. However, the text of the article shows the story is more complex than it may seem at first: the vaccine is still a trial, and this patient was the first one to be tested. The text states, “The drug still has a long way to go, but Knutson said it’s promising and is helping show shades of a future that doctors have been working toward.” We concur that the title is sensationalist, since it leads readers to conclude something that is not yet realistic. This same story, with the same or remarkably similar titles, was reproduced thirty-two times in our sample.

As an example of “misleading content”, we can mention the news story whose title is “Black women are over 6 times more likely to get breast cancer from hair dye and relaxers- New study finds” (145,238 shares). However, this study has a serious limitation, as pointed out by an epidemiologist: “The Sisters Study is a good prospective cohort study—but women were recruited to the study because they had a sister with breast cancer, so the conclusions wouldn’t necessarily hold true for women in the wider population, hence the need for further confirmation.” [36] This type of misinformation can cause the spread of unnecessary fear among the lay audience [37].

The most shared “content types” in the sample of this study were “real-life stories” and “solidarity” (67.69%). This seems to indicate a public preference in relation to these themes. Another study which examined Brazilian Facebook pages about cancer shows similar findings; on most pages, content related to “Solidarity”, “Anniversaries” and “Testimonies or real-life stories” was among those with the most engagement on this social media [38]. To illustrate, the most shared articles classified as “real-life story” in our sample include the following: “Loyal boyfriend who stuck with girlfriend during breast cancer proposes on her last day of chemo” (734,482 total shares) and “Dad with breast cancer 'rejected from support groups because he's a man'” (640,106 total shares). The most shared “solidarity” stories are “DeAngelo Williams Pays for 500 Mammograms after Mom Dies of Breast Cancer” (62,836 total shares) and “North Charleston Police Department goes pink to help fight breast cancer” (61,769 total shares). These stories focus on the daily life, or personal narratives of cancer patients, their family members, or friends. There is no educational or awareness objective regarding risk factors and prevention of breast cancer. One of the stories mentions mammography, but the text does not provide more details about who should undergo the exam, and when it should be scheduled.

Only 5.08% of the articles in our sample address prevention, and 19.7% mention early detection. Extensive literature highlights the importance of adopting habits that help prevent breast cancer, such as limiting alcohol consumption, not smoking, maintaining a healthy weight, and being physically active [39–41]. It is also extremely important that the population is well informed regarding the importance of early diagnosis, including the symptoms of the disease, as screening is the most efficient way to diagnose breast cancer at an early stage and thus decrease mortality [2]. Moreover, a study conducted in Hungary revealed most respondents were unaware of the fact that breast cancer self-examination should be initiated two decades earlier than mammography, when women turn twenty years old [42]. The lack of knowledge and awareness on breast cancer is also an identified concern in a number of highly populated countries, such as Ethiopia [43], Nigeria [44], and Brazil [45]. A systematic review of breast cancer screening discourse on social media [46] indicates there is a substantial presence of unscientific statements shared by lay individuals about the topic, such as “mammography causes breast cancer”, or “breast cancer can be prevented by organic food”. This type of misinformation is dangerous, as it can discourage women from scheduling the screening exam.

It is interesting to note that, in our sample, there is a decrease in news stories that address risk factors in October, the Breast Cancer Awareness Month. On the other hand, there is an increase in stories regarding solidarity. Although the stories that narrate solidarity attitudes are more popular (high number of shares on social networks), we believe it is also necessary to address issues related to prevention, risk factors and early diagnosis more emphatically throughout this month. A study that examined Google searches for the terms "mammography" and "breast cancer" over a five year period showed remarkable peaks every October [47], which reveals a growing, although temporary, interest of the population in the subject. Another study that investigated Twitter messages during the awareness month concluded most content on this SNS does not address any type of prevention strategies, and it is essentially used as a one-way communication tool [48].

## Limitations

This study has some limitations that need to be considered. The first aspect is the limited number of investigated news stories, as we do not have enough resources to analyse qualitatively thousands of articles without compromising the excellence of the process. Due to this, we have no way of knowing whether the result of the content analysis of the entire corpus will be the same as the analysis of the sample selected for this study. A second limitation is the fact that our sample is limited to stories in English. If we analysed other languages, we might have discovered differences in the topics covered and in the credibility of the news. Therefore, we believe it is not possible to generalize the results observed in this article to all languages and cultural settings.

## Conclusions

The study revealed that, although the volume of verified, evidence-based content is moderately greater in our sample than misinformation, unscientific articles are shared 3.29 times more, on average. In an environment in which everyone can produce content without any type of filter or quality control, public understanding of medical research and advances has never been more paramount [49]. We consider it is of great importance to combine content of higher popularity, such as real-life stories, with subjects that can make a difference in patients’ lives, such as early detection, breast cancer symptoms and disease prevention strategies. The same should be done during Pink October, when there is an increase in internet searches on the topic. Speaking about solidarity while addressing the importance of screening exams can be a good strategy. We believe our findings may be useful to assist in the development of online health communication strategies in breast cancer. Doctors, scientists and health journalists can expand the dialogue with the lay public about breast cancer, helping to counteract online misinformation.

## Data Availability

The datasets used and/or analysed during the current study are available from the corresponding author on reasonable request.

## Abbreviations

SNSs: Social networking sites
COVID-19: 2019 Novel coronavirus
SPSS: Statistical Package for the Social Sciences
P: P-value
